# The Influence of Personality Traits on Driving Behaviors in Preclinical Alzheimer Disease

**DOI:** 10.1093/geroni/igaf122.3383

**Published:** 2025-12-31

**Authors:** David Carr, Andrew Aschenbrenner, Ganesh Babulal

**Affiliations:** Washington University in St. Louis, St Louis, Missouri, United States; Washington University in St. Louis, St. Louis, Missouri, United States; Washington University School of Medicine in St. Louis, SAINT LOUIS, Missouri, United States

## Abstract

Alzheimer disease (AD) has a long preclinical phase in which AD pathology is accumulating without detectable clinical symptoms. It is critical to identify participants in this preclinical phase as early as possible since treatment plans may be more effective in this stage. Monitoring for changes in driving behavior, as measured with GPS sensors, has been explored as a low-burden, easy to administer method for detecting AD risk. However, driving is a complex, multi-faceted process that is likely influenced by other factors that may change in preclinical AD, including personality traits. In this study, we examine the moderating influence of neuroticism and conscientiousness on longitudinal changes in driving behavior in a sample of 203 clinically normal older adults who are at varying risk of developing AD. Our results indicate that neuroticism moderated rates of change in the frequency of speeding as well as the number of trips taken at night. Conscientiousness moderated rates of change in typical driving space. We interpret these findings within established changes in personality traits in preclinical AD and suggest that studies of longitudinal changes in naturalistic driving behavior need to accommodate interpersonal differences, including personality. Strengths and limitations of this study are also noted. Future studies should explicitly establish how much benefit or risk is provided by including personality traits in predictive models of AD progression.

